# Genomic Characteristics of Colistin-Resistant *Salmonella* *enterica* subsp. *enterica* Serovar Infantis from Poultry Farms in the Republic of Serbia

**DOI:** 10.3390/antibiotics9120886

**Published:** 2020-12-10

**Authors:** Branko Jovčić, Katarina Novović, Brankica Filipić, Maja Velhner, Dalibor Todorović, Kazimir Matović, Zoran Rašić, Sonja Nikolić, Ferenc Kiškarolj, Milan Kojić

**Affiliations:** 1 Institute of Molecular Genetics and Genetic Engineering, University of Belgrade, 11010 Belgrade, Serbia; bjovcic@bio.bg.ac.rs (B.J.); katarinanovovic@imgge.bg.ac.rs (K.N.); mkojic@imgge.bg.ac.rs (M.K.); 2 Faculty of Biology, University of Belgrade, 11000 Belgrade, Serbia; 3 Faculty of Pharmacy, University of Belgrade, 11000 Belgrade, Serbia; brankica.filipic@pharmacy.bg.ac.rs; 4 Scientific Veterinary Institute “Novi Sad”, 21000 Novi Sad, Serbia; dalibor@niv.ns.ac.rs; 5 Veterinary Specialized Institute “Kraljevo”, 36000 Kraljevo, Serbia; matovic@vsikv.com; 6 Veterinary Specialized Institute “Jagodina”, 35000 Jagodina, Serbia; vsij.rasic@gmail.com; 7 Veterinary Specialized Institute “Niš”, 18000 Niš, Serbia; sonjavsinis@gmail.com; 8 Veterinary Specialized Institute “Subotica”, 24000 Subotica, Serbia; kiskarolj.ferenc@vsisu.co.rs

**Keywords:** *Salmonella*, colistin, quinolones, resistance, WGS, *pmrB*

## Abstract

The antimicrobial susceptibility testing was conducted on 174 single isolates from poultry farms in Serbia and it was determined that seven *Salmonella* spp. were multidrug resistant. Sixteen serotypes were detected, but only serotype Infantis confirmed reduced susceptibility to colistin. Seven colistin resistant *Salmonella* Infantis were studied in detail using the WGS approach. Three sequence types were identified corresponding to different epizootiology region. The isolate from the Province of Vojvodina 3842 and isolates from Jagodina (92 and 821) are represented by the sequence type ST413 and ST11, respectively. Four isolates from Kraljevo are ST32, a common *S.* Infantis sequence type in humans, poultry and food. The fosfomycin resistance gene *fosA7* in isolate 3842 and the *vgaA* gene in isolate 8418/2948 encoding resistance to pleuromutilins were reported for the first time in serovar Infantis. The changes in relative expression of the *phoP*/*Q*, *mgrB* and *pmrA/B* genes were detected. Single nucleotide polymorphisms of the *pmrB* gene, including transitions Val164Gly or Val164Met, and Arg92Pro are described. Analyses of quinolone resistance determining region revealed substitutions Ser83Tyr in GyrA protein and Thr57Ser and Ser80Arg in ParC protein. Based on WGS data, there are two major clusters among analyzed *Salmonella* Infantis isolates from central Serbia.

## 1. Introduction

As reported by the World Health Organization (WHO), salmonellosis is taking third place among foodborne diseases in humans, causing death. In European Union, *Salmonella* Infantis has been ranked among five most prevalent infection-causing serovars in humans [1]. Unlike *S.* Enteritidis, *S.* Infantis serovar tends to develop multidrug resistant phenotype more often. It has been recognized as a persistent clone, causing long lasting contamination at poultry farms in Europe and Japan [2,3]. In addition, an *S.* Infantis epidemic clone was identified in a rehabilitation center of an oncology clinic in Germany, indicating that poultry and/or kitchen contamination may present a possible reservoir of infection [4]. Such contamination ability could be considered a result of vast diversity of virulence mechanisms, like those encoded by genes found on mega plasmids pESI in *S.* Infantis isolates in Israel [5]. It was also proposed that mutations in the key regulatory virulence genes or mobility of genetic elements in isolates from patients suffering from persistent infection may lead to a prolonged shedding of *Salmonella* in humans by facilitating clonal competition [6]. Multidrug resistant *S.* Infantis strains carrying large conjugative plasmid with *tetA* gene and integron 1 were identified in Hungary. These isolates belong to various, but closely related pulsotypes, including the new B2 type established in humans and poultry hosts, not only in Hungary, but also in other European countries [7,8]. The mega plasmid pESI was found in *S.* Infantis isolates from the USA, Latin America, Japan, and Russia as well [9]. Therefore, *S.* Infantis is an important serovar in human medicine and food producing animals frequently serve as a vehicle of transmission. This serovar is difficult to eradicate at poultry farms due to lack of serovar specific vaccines. Not only that *S.* Infantis have the ability to develop multiple mechanisms of antimicrobial resistance, but some strains produce extended-spectrum-β-lactamase enzymes [10,11,12,13]. It is also alarming that plasmid mediated resistance to colistin was detected recently in *S.* Infantis isolates from broilers in Italy [14]. Since colistin is the last resort antibiotic for the therapy of intestinal infections in humans, it is important to monitor resistance to this agent in isolates from food producing animals all around the globe. Approximately 20% of *S.* Infantis isolates from humans, poultry, and food from Serbia from 2008–2011 were resistant to ciprofloxacin due to mutations in *gyrA* and *parC* genes. At the time, the resistance to colistin was not investigated [15]. Therefore, in 2018 we continued antimicrobial susceptibility testing of *Salmonella enterica* isolates from poultry farms and determination of colistin minimal inhibitory concentration was included in the study. In addition, the whole genome sequencing (WGS) was used for in-depth analysis of resistance and virulence genes in colistin-resistant S. Infantis strains, as well as for establishing genetic relatedness between those strains.

## 2. Results and Discussion

Due to public health significance of resistance to polymixins in Gram-negative bacteria, a research was undertaken to evaluate resistance to antibiotics, including colistin, in *Salmonella* spp. isolates from poultry farms in Serbia. *S.* Infantis is a well-established clone in poultry flocks all around the world due to extremely persistent infections in poultry and contamination of the environment [2,5,16]. On the other hand, colistin is widely used to treat intestinal infections of poultry in Serbia, in spite of constant warnings to avoid therapy with antibiotics critically important for human medicine.

### 2.1. Antimicrobial Susceptibility Testing

Antimicrobial resistance profiles of isolates respective to resistotypes included in this study are listed in Table 1. Altogether, unrelated mechanisms conferred resistance to three or more classes of antibiotics in nine *Salmonella* spp. isolates. Those were six isolates of serovars Infantis, and one isolate of serovar Stanley, serovar Kotbus and Typhimurium. Multidrug resistance in *Salmonella* spp. is often detected in well-established worldwide clones such as *S*. Typhimurium definite phage type 104 (STDT104) or *S*. Kentucky, sequence type 198, which are frequently resistant to ciprofloxacin [17,18]. However, in this work, only 1 out of 15 *S*. Typhimurium isolates was resistant to multiple antibiotics (AMP, SA, TMP, and SXT). It is interesting to note that *S*. Typhimurium isolates from poultry in Serbia are susceptible to antibiotics and that this serovar is rarely detected in other food-producing animals. The lack of surveillance and monitoring of antimicrobial resistance in veterinary medicine in Serbia (presently) or the use of less sensitive methods for *Salmonella* detection in some veterinary laboratories could explain the low numbers of *S.* Typhimurium isolates in food-producing animals. Out of the seven *S.* Infantis isolates included in the WGS analysis four were both multidrug-resistant and with reduced susceptibility to colistin (MICs from 4 µg/mL to >16 µg/mL), and three were susceptible to 14 antibiotics but were resistant to colistin only. Therefore, the acquired resistance to colistin may be easily overlooked in isolates susceptible to some other antibiotics.

### 2.2. Molecular Typing of Colistin-Resistant S. Infantis Isolates

Genomic DNA of seven *S.* Infantis isolates with reduced susceptibility to colistin were sequenced using Illumina HiSeq 2500 platform. The contig dataset was used to determine functional analysis. MLST analysis of seven *S.* Infantis colistin-resistant isolates resulted in identification of three sequence types from Serbian poultry farms, ST32 (isolates 1371/1, 9181/1, 8418/2948, and 9520/2), ST11 (isolates 92 and 821) and ST413 (isolate 3842) (Table 2). It was determined that ST32 is the most frequent sequence type in serovar Infantis [19,20,21,22]. The scarcity of ST11 and ST413 related research in *S.* Infantis, could be related either to rare occurrence or less significant pathogenic potential.

### 2.3. Resistome of Colistin-Resistant S. Infantis Isolates

Due to the growing problem of antibiotic resistance in Gram negative bacteria, polymyxin E (colistin) and polymyxin B are used as valuable antibiotics for the therapy of complicated infections in humans. However, bacteria may develop acquired resistance to polymyxins utilizing different levels of resistance, mostly toward adaptive mechanisms [23]. Among determinants of colistin-resistance, the two-component regulatory (TCS) systems, including the PmrAB regulon, represent the common mechanism important for the pathogenesis of *Salmonella* spp. [24]. Cationic antimicrobial peptides, such as polymixin, bind to LPS in the cell membranes and modify their permeability, causing the death of the bacterial cells. However, mutations in *pmrA*/*pmrB* genes induced by colistin reduce the negative charge of the LPS, which results in lower susceptibility to this antibiotic in *Salmonella enterica* [25]. Another important mechanism of resistance to colistin is mediated by the plasmid-borne the *mcr1* gene. This was first discovered during routine monitoring in China by Liu et al. (2015) [26] in commensal *Escherichia coli* isolates from food-producing animals. The plasmid-mediated the *mcr1* gene was transferable to other bacterial species. Further investigation revealed mobile resistance to colistin in *E. coli* isolates from patients as well. After the initial report from China, several new variants of the *mcr1* gene (*mrc1*-*mcr9*) were further identified all over the world [23]. These discoveries are alarming since mobile plasmids may carry genes coding for resistance to several antibiotics and be transferred by the mechanism of co-selection [27]. In addition, it was shown that the *mcr* gene can become a transposon-mediated gene or may also be found in the chromosome of *E. coli* [23]. The overuse of colistin in the livestock industry, especially in developing countries, largely contributes to the spread of colistin-resistant bacteria. Therefore, the misuse of old antibiotics such as colistin is creating new problems in a situation that is already difficult in both human and veterinary medicine due to the emergency of pan-resistant bacteria.

Whole-genome sequencing data of seven colistin-resistant isolates were subjected to in silico analysis for the presence of resistance conferring genes and mutations. Among colistin-resistant isolates from Serbian poultry farms, only those from ST32 group had mutations in the *pmrB* gene, resulting in Val164Met (isolates 9181/1 and 9520/2), Val164Gly (isolate 1371/1), and Arg92Pro (isolate 8418/2948) amino acid substitutions. Mutations in the *phoPQ*, *pmrA*, and *mgrB* genes were not found in the analyzed genomes (Table 2). Previously, high mutation rate in *Salmonella* Typhimurium LT2, especially in the *pmrB* gene, resulting in 22 amino acid substitutions at 17 different positions, were detected in mutants obtained by in vitro cultivation in medium supplemented with colistin [25], but none of the mutations were identical to the one observed in this paper. Although we cannot attribute colistin-resistance phenotype of the analyzed isolates to these substitutions, since we do not have experimental confirmation, there is a possibility that they result in resistance phenotype according to extraordinary divergence of substitutions in the PmrB protein described so far. Additionally, data from *S.* Infantis are lacking, thus limiting the possibilities for comparison.

Resistance to quinolones in *Salmonella* spp. is represented by at least three important mechanisms, which include mutations within quinolone resistance-determining regions (QRDR), plasmid mediated resistance (PMQR) and antibiotic efflux driven by efflux pumps. Analysis of quinolone resistance-determining region revealed mutations in the *gyrA* and *parC* genes of all ST32 isolates and in the *parC* gene for ST413 isolate. Four isolates of ST32 group (57.14% of the total number) had mutation in the *gyrA* gene, resulting in Ser83Tyr amino acid transitions. Mutation resulting in Thr57Ser transition was found in the *parC* gene of five isolates from both ST32 and ST413 groups (71.43% of total number) and Ser80Arg was found only in the *parC* gene of one isolate (14.29%). Mutations in *gyrB* and *parE* genes were not found in the analyzed genomes (Table 2). Previously, amino acid substitutions Ser83Tyr in GyrA and Ser80Arg in ParC were found in fluoroquinolone resistant *S.* Infantis from Serbia [15]. All of the mutations detected are often found in other non-typhoidal *Salmonella enterica* as indicated in the research paper of Neuert et al. [28] and Monte et al. [29]. In this paper, the PMQR genes were not detected. Applying the WGS approach, RND, MFS, ABC, and MATE transporters in colistin-resistant *S.* Infantis were identified. These transporter systems play a role in extruding various harmful compounds from bacteria, contributing in establishing long-lasting infection in hostile environments [30]. Specific alterations at global regulators of the efflux pump belonging to RND family were found to induce increased resistance to fluoroquinolones in mutants other than *S.* Typhimurium by Kehrenberg et al. [31].

The aminoglycoside acetyltransferase gene *aac(6′) Iaa* confers resistance to tobramycin, kanamycin and amikacin and this gene was found in *S.* Infantis isolates from this paper (Table 2, Supplementary Tables S1 and S2). Previously, this chromosomal gene was identified in *Salmonella* Typhimuirum LT2, but it does not have substantial evolutionary advantage and is of less clinical significance [32]. Resistance to β-lactam antibiotics was encoded by the *bla*_TEM-1B_ gene in isolate 9520/2 or *ampC*-like gene in all seven isolates. In isolate 3842, *bla*_oxa-22_ gene was identified by WGS. Resistance to bacitracin was encoded by the *bacA* gene in all isolates included in genetic analysis. Interestingly, for the first time, a fosfomycin resistance gene *fosA7* was found in an *S.* Infantis isolate 3842 (Table 2, Supplementary Table S1). This gene was for the first time described by Rehman et al., [33] in *S.* Heidelberg isolates from broilers in Canada, while in Brazil the *fosA7* gene was detected in a single *S.* Brandenburg isolate and two isolates of *S.* Heidelberg [29]. The product of the gene is a glutathione S-transferase metalloenzyme with significant potency of transfer via plasmids or other mobile genetic determinants on chromosome. Moreover, resistance to fosfomycin is driven by the use of this antibiotic in human and veterinary medicine and probably has accommodated in more *Salmonella* serovars than previously established [33]. The *vgaA* gene encoding resistance to pleuromutilins by efflux mechanism is hosted on plasmid in methicillin resistant *Staphylococcus aureus* ST398 [34,35]. This gene is not commonly found in *Salmonella* spp. [36], but in this paper it was detected in *S.* Infantis isolate 8418/2948. However, the *vgaC*, streptogramin A resistance gene was detected frequently in *S.* Kentucky isolates and in a single *S.* Typhimuirum isolate from broiler chicken farms in British Columbia, Canada, by Maguire et al. [37]. Resistance to tetracycline is a frequent phenotype in *S.* Infantis and it was not surprising that four out of seven *S.* Infantis isolates expressed resistance to this antibiotic by utilizing the efflux mechanism encoded either by *tetA* gene (1371/1 and 1981/1) or by *tetA* and *tetK* genes (8418/2948 and 9520/2).

### 2.4. Plasmidome Analysis

Plasmid contigs were found only in *S.* Infantis ST11 (isolate 92). pMLST profile was IncF RST, with FIB_22 and FIIS_1 alleles identified. FIB_22 allele was identified within NODE_36 of isolate 92 genomic sequence, which shared 98.91% of identity with plasmid pSJUTF10978 (GenBank CP015525.1; region 2595–49146 bp) of *S.* Enteriditis strain SJTUF10978. FIIS_1 allele was identified within NODE_64 of isolate 92 genomic sequence, which shared 100% identity with plasmid pPT1-1 (GenBank CP043434.1; regions 11189–11370 bp and 13154–23496 bp) of *S.* Enteriditis strain PT-1. However, replicon typing PCR developed by Caratolli et al., [38] has revealed that *S.* Infantis isolates 9520/2, 9181/1, 8418/2948, and 1371/1 possess IncP plasmids while isolate 821 has plasmid IncFIIA. In addition, the PCR replicon typing system confirmed the existence of the IncFIIA plasmid in isolate No. 92. This result could indicate the high prevalence of extrachromosomal DNA in our isolates, and thus the possibility of horizontal gene transfer potential and history. In the recent research of McMillan et al. [39], it was established that 157 out of 193 *Salmonella* spp. isolates had at least one plasmid and that many of them carried significant number of resistance genes. All of the isolates assessed in their research were from the NARMS isolate collection obtained from food animals and a few *S.* Heidelberg strains from humans

### 2.5. Virulence and Salmonella Pathogenicity Islands

*In silico* search for virulence determinants revealed presence of nonfimbrial and fimbrial adherence determinants, genes for magnesium uptake, *phoPQ*, macrophage inducible genes (*mig-5* or *mig-14*), TTSS encoded by SPI-1, SPI-2, and both TTSS-1 and TTSS-2 translocated effectors. Besides those general factors, the *rck* serum resistance factor, *spvABR* and ACE T6SS were present in genomes of isolates belonging to sequence type ST11 (Supplementary Table S2). Isolates belonging to ST32 were characterized by presence of AFA-I adhesion factor, K88 fimbriae, type IV pilli, iron acquisition and uptake system. Within this group, isolates 9181/1 and 8418/2948 had beta-hemolysin and elements of immune evasion (capsule, LPS glucosylation and polyglutamic acid capsule (Supplementary Table S2). Isolate 3842 (ST413) was the only one with *ibeB* gene encoding for invasion of brain endothelial cells factor. The isolate 8418/2948 from Kraljevo possessed the highest number of virulence genes, compared to other *S.* Infantis isolates. This included gene encoding beta hemolysin, acid resistance gene, elastin binding protein, polar flagella, *Streptococcus* plasmin receptor, type IV pilli, anaerobic respiration gene, several enzymes, immune evasion genes, and iron acquisition gene *fagC*.

The epidemiology and pathogenesis of *S.* Infantis has been studied comprehensively by several research groups. It was discovered that the emerging *S.* Infantis clone in Israel possesses a large mosaic plasmid pESI with resistance and virulence genes, including chaperon-usher fimbriae operons (K-88 like), involved in host tropism and pathogenicity. This new strain has replaced classical *S.* Infantis within a few years in Israel and, surprisingly, the large plasmid increased the fitness of the strain [5]. *S.* Infantis with the endemic multiresistant plasmid pSI54/04 was also detected in Hungary, but the plasmid transfer to pre-emergent strain did not increase virulence in one day old chicken after challenge experiments [40]. Important virulence factor in non-typhoidal *Salmonella* serovars is the Typhi colonization factor Tcf due to the role of this fimbria in pathogenicity. In *S.* Infantis, Tcf is responsible for increased colonizing capacity of mouse intestine, while this gene expression was lower in *S.* Swarzengrund and *S.* Heidelberg compared to *S.* Infantis [41]. Seven isolates of *S.* Infantis from this paper also possess Tcf virulence factors and five isolates (1371-1, 8418-2948, 9502-2, 9181-1, and 3842) possess adherence factor K88, suggesting that those properties may play a role in persistent intestinal infection of poultry in Serbia as well (Supplementary Table S2).

*Salmonella* pathogenicity islands (SPI) harbor a variety of virulence genes that are required for interaction with hosts and they are differently arranged through the genome of *Salmonella*. These pathogenicity islands carry genes that are involved in host invasion, systemic infection, replication within macrophages, macrophage apoptosis mechanism, and they carry genes that encode effector proteins of type III secretion system T3SS and T2SS, which are important for pathogenicity [42,43].

The array of SPI in *Salmonella* Infantis is presented in Table 3. All isolates analyzed in this paper possess SPI-1, SPI-2, SPI-5, SPI-13, SPI-14, and C63PI, except isolates 3842 and 8418/2948. However, in seven isolates from this paper, SPI-6 with genes responsible for encoding protein transport system was not detected. SPI-13 is a nutritional fitness locus, and it was discovered that this locus induces metabolic functions by mediating utilization of D-glucuronic acid and tyramine, the elements presenting the source of carbon and nitrogen [44]. The SPI-14 was found in *S.* Infanits from this paper as well (Table 3), while it is absent in human serovars Typhi and Paratyphi A [43]. In a mouse model of infection, *S.* Typhmuirum SPI-14 deletion mutants responded by reducing virulence in mice after oral infection and reducing invasion of epithelial cells. In wild type isolates, this locus activates expression of SPI-1 genes in low oxygen levels and as a response to other specific signals triggered when *Salmonella* reach distal ileum during infection cycle [43].

According to CRISPRCasFinder, all isolates harbor CRISPR-Cas system Class 1 with 29 bp repeats (consensus repeat ID R2887 CRISPRdb). However, 8 *cas* genes were detected in isolate 92 (compared to 9 detected in other isolates) and only one *cas* gene in isolate 3842 (Cas3_0_IE) (Supplementary Table S3).

### 2.6. Pan-Genome Analysis

Pan-genome reflects the total number of genes that are present in a given dataset and the main goal of pan-genome analysis is genomic comparison of different isolates of the same species [45]. Pan-genome analysis revealed a total of 5618 gene clusters, which were separated into the core genome, comprised of 3802 genes (3802 hard core and 0 soft core genes) and accessory genome containing 1195 genes in the shell and 621 genes in the cloud (Supplementary Figure S1). The core genome of seven *S.* Infantis isolates revealed that the analyzed genomes are phylogenetically related, as they share a high number of common genes (Supplementary Figures S1 and S2). Pan-genome analysis showed high correlation between sequence type and present accessory genes (Supplementary Figure S2). Besides that, isolate 3842 belonging to underrepresented sequenced type ST413 in *Salmonella* spp. had the highest number of unique genes among the analyzed genomes (Supplementary Figure S2). Results of BPGA, COG, and KEGG analyses revealed that accessory genes, which include genes not present in all genomes, are mostly involved in cellular metabolism (Supplementary Figure S3), thus probably providing metabolic advantages to different isolates in different conditions [46]. Moreover, the genes of importance for infectious diseases were mostly identified within the unique genes of the pan-genome (Supplementary Figures S1–S3).

### 2.7. Expression Analysis of Colistin-Resistance Associated Genes

**Figure 1:**
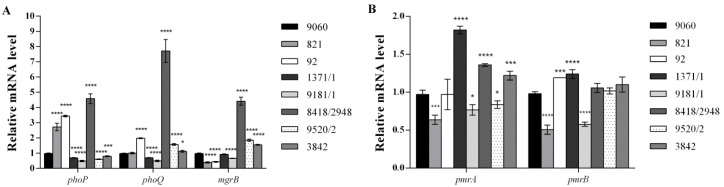
Relative expression level of colistin-resistance associated genes from tested colistin-resistant *S.* Infantis isolates. (**A**) Relative expression level of the *phoP*, *phoQ*, and *mgrB* genes. (**B**) Relative expression level of the *pmrA* and *pmrB* genes. All expression results were normalized relative to the *gyrB* gene by the 2−ΔΔCt method. Values are the means from results obtained in triplicate. Error bars represent the standard deviation of the mean value. One way ANOVA followed by Tukey’s post hoc test was used to compare the results obtained for colistin-resistant isolates to those for colistin-susceptible isolate *Salmonella* Infantis 9060((* *p* < 0.05, *** *p* < 0.001, and **** *p*< 0.0001).

In order to establish molecular mechanism responsible for colistin resistance in the tested isolates, transcription analysis of the following genes was performed: *phoP*, *phoQ*, *mgrB*, *pmrA*, and *pmrB*. *phoPQ* mRNAs level was statistically increased in isolates 92 and 8418/2948, while it was decreased in isolates 1371/1 and 1981/1 (Figure 1A). It was noticed that in isolate 821 only transcription of the *phoP* gene was elevated and in isolates 9520/2 and 3842 expression of PhoP and PhoQ was opposite (PhoP decreased and PhoQ increased). Transcription of the *mgrB* gene was downregulated in isolates belonging to ST11 (821 and 92) and one ST32 isolate (9181/1), but upregulated in two isolates belonging to ST32 (8418/2948 and 9520/2), as well as ST413 (3842) (Figure 1A). The level of the *pmrA* mRNA was increased in three isolates (1371/1, 8418/2948, and 3842) while expression of the *pmrB* gene was upregulated in isolates 92 and 1371/1 (Figure 1B). Although variations in mRNA levels of the analyzed genes in different isolates could be confusing, we should take into account the complexity of this system where PhoPQ and PmrAB signaling systems positively regulate modifications of LPS leading to colistin resistance, but also active PhoPQ induces MgrB expression, which exerts negative feedback on the same regulatory system. Also, clear and unambigous correlation could not be drawn between values of MIC and relative expression of selected genes for isolates included in this study.

## 3. Materials and Methods

### 3.1. Isolates

One hundred and seventy-four single isolates from poultry farms in Serbia were included in the study. Five veterinary institutes from the north to the south of the country participated by providing *Salmonella enterica* isolates. Samples were collected during official routine monitoring program, as stated in the *Official Gazette of the Republic of Serbia*, No. 36: The rulebook for early detection, diagnostics, prevention, suppresion and eradication of particulate *Salmonella* serotypes in poultry flocks. Therefore, the samples were overshoes or feces collected from poultry farms. The following serovars were identified through the study: *S.* Infantis were represented by 51 isolates, *S.* Enteritidis by 78 isolates, *S.* Typhimurium by 15 isolates, *S.* Tennessee by 6 isolates, *S.* Seftenberg by 5 isolates, *S.* Mbandaka by 3 isolates, *S.* Newport, *S.* Hadar, *S.* Kottbus, and *S.* Yoruba by 2 isolates while *S.* Bovismorbificans, *S.* Nitra, *S.* Stanleyville, *S.* Virchow, *S.* Napoli, *S.* Stanley and *S.* Thompson were represented by 1 isolate each. One isolate was typed as group E (O:19). Except for *S.* Infantis, and *S.* Enteritidis, all other serovars were sent to the Institute of Public Health of Serbia, Reference Laboratory for *Salmonella*, *Shigella*, *Vibrio cholere*, and *Yersinia enterocolitica*, Belgrade, Serbia for serological identification. Results of antimicrobial susceptibility testing including determination of colistin MIC are provided in Table 1.

### 3.2. Antimicrobial Susceptibility Testing

Susceptibility testing was done by disk diffusion method. Interpretation of the results was performed according to recommendation of the Clinical and Laboratory Standards Institute CLSI (documents number M07-A10 and M100-S25) [47,48]. The following disc by BioRad (Marnes-la-Coquette, France) were used: ampicillin 10 μg (AMP), amoxicillin/clavulanic acid 20 μg + 10 μg (AMC), chloramphenicol 30 μg (CHL), ciprofloxacin 5 μg (CIP), gentamicin 10 μg (GEN), nalidixic acid 30 μg (NAL), streptomycin 10 μg (STR), sulfonamides (sulfisoxazole-like compound) 300 μg (SA), tetracycline 30 μg (TET), trimethoprim/sulfamethoxazole 1.25/23.75 μg (SXT), trimethoprim 5 μg (TMP), cefpodoxime 10 μg (CPD), cefotaxime 30 μg (CTX), and ceftazidime 30 μg (CAZ). For the quality control the *Escherichia coli* ATCC 25922 was included in the study, each day when antimicrobial susceptibility testing was performed. Multiresistance was designated if isolates were resistant to three or more antibiotics of different classes [49]. *S*. Enteritidis were susceptible to antibiotics included in antimicrobial susceptibility testing.

### 3.3. Minimal Inhibitory Concentrations for Colistin

The minimal inhibitory concentration (MIC) analyses were done according to Gwozdzinski et al. (2018) [50] by broth microdilution method. Following their protocol, Mueller Hinton broth was purchased by (Sigma-Aldrich, Darmstadt, Germany, product number 70192) and supplemented with calcium chloride dehydrate (Carl Roth, Karlsruhe, Germany, product number 5239.3), (Gwozdzinski et al., 2018). Colistin sulfate was from Sigma-Aldrich, (Darmstadt, Germany). For the quality control the *E. coli* ATCC 25922 and *E. coli* NCTC 13846 were included on each plate. The interpretation of MIC values for ATCC 25922 control strain recommended by European Committee on Antimicrobial Susceptibility Testing (EUCAST) range from 0.25–2 mg/L. The NCTC 13846 results in MIC of 4 mg/L. The MIC analysis of *Salmonella* spp. were interpreted according to EU Directive 2013/652/EU [51], which recommends the following resistance break point R > 2 mg/L. All isolates presented with the MIC ≥ 2mg/L were additionally inoculated on Mueller Hinton agar supplemented with 2mg/L of colistin sulfate for confirmation of the growth or opposite [52]. *S.* Enteritidis was excluded from the MIC analysis because clinical breakpoint of colistin for this serovar is not established [53].

### 3.4. Whole Genome Sequencing and Genome Analyses

Genomic DNA of seven *S.* Infantis isolates was sequenced using Illumina HiSeq (MicrobesNG, IMI-School of Biosciences, University of Birmingham, Birmingham, UK) and the quality of each genome was checked using FastQC [54]. Genome sequences were further analyzed for the presence of genes encoding virulence factors using virulence factor database (VFDB, http://www.mgc.ac.cn/VFs/main.htm) [55]. Genetic determinants of antibiotic resistance were determined using The Comprehensive Antibiotic Resistance Database (CARD, https://card.mcmaster.ca/), ResFinder 3.1 (www.genomicepidemiology.org). In addition, presence of resistance-conferring mutations in QRDR and *pmrAB*, *phoPQ*, and *mgrB* genes was confirmed using DNA Strider with corresponding genes of *S.* Infantis 1326/28 (GenBank GCA_000953495.1), which were used as a negative control. Plasmidome (PlasmidFinder 2.0), plasmid MLST (pMLST 2.0), multilocus sequence typing (MLST 2.0) and the presence of *Salmonella* pathogenicity islands (SPIfinder 1.0) were analyzed using databases of the Center for Genomic Epidemiology (www.genomicepidemiology.org). Additionally, CRISPRs and cas genes were detected using the CRISPRCasFinder program (https://crisprcas.i2bc.paris-saclay.fr/CrisprCasFinder/Index). Draft genome sequences of seven *S.* Infantis isolates have been deposited at the NCBI GenBank database under accession numbers JAAGKW000000000-JAAGLB000000000 and JAALLI000000000.

### 3.5. Bacterial Pan-Genome Analysis

Pan-genome analysis approach was used in order to compare genomes of seven *S*. Infantis isolates. Genome sequences were firstly annotated using Prokka (version 1.13) and obtained annotated GFF files were further clustered by Roary (version 3.13.0) into core genes (selection threshold for hard core genes was presence in >99% of the isolates and for soft core genes threshold was presence in 95–99% of isolates) and accessory genes (further subdivided into shell genes— present in 15–95% of isolates; and cloud genes—present in less than 15%). Phylogenetic tree was created by Roary and visualized by Phandango. Genomic diversity among seven *S.* Infantis isolates and identification of strain specific features were determined using BPGA tool.

### 3.6. Transcriptional Analysis by Reverse Transcription Quantitative PCR (RT-qPCR)

Isolation of the total RNA from bacterial cells, DNase I treatment and reverse transcription were done as previously reported [56] In brief, colistin-resistant *S.* Infantis isolates were incubated in Luria–Bertani (LB) broth supplemented with 2 µg/mL of colistin sulfate (Sigma-Aldrich) at 37 °C with shaking overnight. The same overnight cultures were used for isolation of DNA for genome sequencing and for further RNA isolation experiments. RNA was isolated from those overnight cultures diluted in the fresh LB broth supplemented with colistin sulfate (2 µg/mL) after reaching OD_600_ value of 0.5. RT-qPCR was used for determination of listed genes transcription level: *phoP*, *phoQ*, *mgrB*, *pmrA*, and *pmrB*. Primers used in RT-qPCR are listed in Supplementary Table S4. RT-qPCR was performed with a KAPA SYBR Fast qPCR Kit (KAPA Biosystems, Wilmington, MA, USA) in a 7500 Real Time PCR System thermocycler (Applied Biosystems, Thermo Fisher Scientific, Waltham, MA, USA). Normalization was done against the *gyrB* gene using the ΔΔ*CT* method (relative). The obtained values were then normalized against results for colistin-susceptible *S.* Infantis isolate 9060. RT-qPCR experiments were done in triplicate.

### 3.7. Statistical Analysis

All results are represented as mean values ± standard deviations. One way ANOVA, followed by Tuckey’s post hoc test was used to compare differences in results obtained for colistin-resistant isolates and colistin-susceptible *S.* Infantis isolate 9060. Values at *p* < 0.05 were considered to be statistically significant.

## 4. Conclusions

In this paper, resistance to fluoroquinolones in *S.* Infantis was not detected, perhaps due to the more responsible use of enrofloxacin antibiotic in the past few years. However, the resistance to nalidixic acid is still worrying, indicating that prudent use of antibiotics must continue in poultry industry in Serbia. Fluctuations in *phoPQ*, *pmrAB*, and *mgrB* mRNA levels that are usually associated with colistin-resistance phenotype were described. Additionally, mutations leading to amino acid susbtitutions were found within the *pmrB* gene. WGS approach has helped identify the peculiar but important genetic differences among *S.* Infantis, which will help with future epidemiological studies worldwide.

## Figures and Tables

**Table 1:** Antimicrobial susceptibility of *Salmonella enterica* isolates from poultry (excluding *S*. Enteritidis), Republic of Serbia.

Number of Isolates with the Distinctive Resistotype	*Salmonella enterica* Serovar Numbers	Resistance Patterns
127	*S.* Enteritidis (78) *S*. Infantis (13), *S*. Typhimurium (14), *S*. Tennessee (6), *S*. Senftenberg (5), *S*. Mbandaka (3), *S*. Yoruba (2), group E (O:19) (1), *S*. Kotbus (1), *S*. Nitra (1), *S*. Napoli (1), *S*. Stanleyvile (1), *S*. Bovismorbificans (1)	S
3	*S*. Infantis (3)	CST
30	*S*. Infantis (28), *S*.Virchow (1), *S*.Thomson (1)	NAL, TET
2	*S*. Newport (2)	AMP, TET
1	*S*. Infantis (1)	SA, TET
2	*S*. Hadar (2)	STR, TET
2	*S*. Infantis (2)	NAL, CST, TET
3	*S*. Kotbus (1), *S*. Infantis (1), *S*. Stanley (1)	AMP, NAL, TET
1	*S*. Typhimurium (1)	AMP, SA, TMP, SXT
2	*S*. Infantis (2)	AMP, NAL, CST, TET, TMP,
1	*S*. Infantis (1)	AMP, GEN, NAL, STR, TET

**Table 2:** Resistome of colistin-resistant *S.* Infantis isolates from Serbian poultry farms.

Isolate	Sequence Type	Resistance Genes	Detected Amino Acid Substitutions
821	ST11	*aac(6′)-Iaa*; *ampC*-like; *bacA*; MATE transporter; RND, MFS and ABC efflux pumps	*/*
92	ST11	*aac(6′)-Iaa*; *ampC*-like; *bacA*; MATE transporter; RND, MFS and ABC efflux pumps	*/*
1371/1	ST32	*aac(6′)-Iaa*; *tetA*; *ampC*-like; *bacA*; MATE transporter; RND, MFS and ABC efflux pumps	*pmrB* V164G *parC* T57S, S80R *gyrA* S83Y
9181/1	ST32	*aac(6′)-Iaa*; *tetA*; *ampC*-like; *bacA*; MATE transporter; RND, MFS and ABC efflux pumps	*pmrB* V164M *parC* T57S *gyrA* S83Y
8418/2948	ST32	*aac(6′)-Iaa*; *tetA*; *tetK*; *vga(A)*; *ampC*-like; *bacA*; MATE transporter; RND, MFS and ABC efflux pumps	*pmrB* R92P *parC* T57S *gyrA* S83Y
9520/2	ST32	*aac(6′)-Iaa*; *tetA*; *tetK*; *bla*_TEM-1B_; *ampC*-like; *bacA*; MATE transporter; RND, MFS and ABC efflux pumps	*pmrB* V164M *parC* T57S *gyrA* S83Y
3842	ST413	*aac(6′)-Iaa*; *bla*_OXA-22_; *fosA7*; *ampC*-like; *bacA*; MATE transporter; RND, MFS and ABC efflux pumps	*parC* T57S

**Table 3:** *Salmonella* pathogenicity islands of seven *S*. Infantis colistin-resistant isolates from Serbian poultry farms according to SPIfinder 1.0 and virulence factor database (VFDB).

*Salmonella* Infantis	MLST Sequence Type	*Salmonella* Pathogenicity Island
821	ST11	SPI-1, SPI-2, SPI-3, SPI-4, SPI-5, SPI-13, SPI-14, C63PI
92	ST11	SPI-1, SPI-2, SPI-3, SPI-4, SPI-5, SPI-13, SPI-14, C63PI
1371/1	ST32	SPI-1, SPI-2, SPI-3, SPI-4, SPI-5, SPI-13, SPI-14, C63PI
9181/1	ST32	SPI1, SPI-2, SPI-3, SPI-4, SPI-5, SPI-13, SPI-14, C63PI
8418/2948	ST32	SPI-1, SPI-2, SPI-3, SPI-5, SPI-13, SPI-14, C63PI
9520/2	ST32	SPI-1, SPI-2, SPI-3, SPI-4, SPI-5, SPI-13, SPI-14, C63PI
3842	ST413	SPI-1, SPI-2, SPI-4, SPI-8, C63PI

## Supplementary Materials

The following are available online at https://www.mdpi.com/2079-6382/9/12/886/s1, Table S1: Resistome prediction for seven colistin-resistant S. Infantis isolates analyzed by the Comprehensive Antibiotic Resistance Database, Table S2: Genes encoding virulence factors from genomes of seven colistin-resistant S. Infantis isolates detected using virulence factor database, Table S3: The CRISPR/Cas loci of seven colistin-resistant S. Infantis isolates detected using CRISPRCasFinder program, Table S4: Primers used in this study, Figure S1: Distribution of the core and accessory genes within pan-genome, Figure S2: Visualization of pan-genome constructed using Roary based on the core and accessory genes showing phylogenetic relatedness of the isolates by blue (present) and white (absent) fragments. At the bottom of the figure the percentage of isolates that have each gene is shown, Figure S3: Pan-genome analysis: pan and core genome curves (A); frequency distribution of gene families within genomes (B); the number of new genes added to each genome (C); the COG and KEGG distribution of the representative proteins in the core, accessory, and unique genome (D and E).

## References

[1] Ferrari R.G., Rosario D.K.A., Cunha-Neto A., Mano S.B., Figueiredo E.E.S., Conte-Junior C.A. (2019). Worldwide epidemiology of *Salmonella* serovars in animal-based foods: A meta-analysis. Appl. Environ. Microbiol..

[2] Hauser E., Tietze E., Helmuth R., Junker E., Prager R., Schroeter A., Rabsch W., Fruth A., Toboldt A., Malorny B. (2012). Clonal dissemination of *Salmonella enterica* serovar Infantis in Germany. Foodborne Pathog. Dis..

[3] Sasaki Y., Ikeda A., Ishikawa K., Murakami M., Kusukawa M., Asai T., Yamada Y. (2012). Prevalence and antimicrobial susceptibility of *Salmonella* in Japanese broiler flocks. Epidemiol. Infect..

[4] Miller T., Brockmann S., Spackova M., Wetzig J., Frank C., Pfeifer Y., Braun P.G., Prager R., Rabsch W. (2018). Recurrent outbreaks caused by the same *Salmonella enterica* serovar Infantis clone in a German rehabilitation oncology clinic from 2002 to 2009. J. Hosp. Infect..

[5] Aviv G., Tsyba K., Steck N., Salmon-Divon M., Cornelius A., Rahav G., Grassl G.A., Gal Mor O. (2014). A unique megaplasmid contributes to stress tolerance and pathogenicity of an emergent *Salmonella enterica* serovar Infantis strain. Environ. Microbiol..

[6] Marzel A., Desai P.T., Goren A., Schorr Y.I., Nissan I., Porwollik S., Valinsky L., McClelland M., Rahav G., Gal-Mor O. (2016). Persistent infection by nontyphoidal *Salmonella* in humans: Epidemiology and genetics. Clin. Infect. Dis..

[7] Nógrády N., Tóth A., Kostyák A., Pászti J., Nagy B. (2007). Emergency of multidrug-resistant clones of *Salmonella* Infantis in broiler chickens and humans in Hungary. J. Antimicrob. Chemother..

[8] Nógrády N., Király M., Davies R., Nagy B. (2012). Multidrug resistant clones of *Salmonella* Infantis of broiler origin in Europe. Inter. J. Food Microbiol..

[9] Bogomazova A.N., Gordeeva V.D., Krylova E.V., Soltynskaya I.V., Davydova E.E., Ivanova O.E., Komarov A.A. (2020). Mega-plasmid found worldwide confers multiple antimicrobial resistance in *Salmonella* Infantis of broiler origin in Russia. Int. J. Food Microbiol..

[10] Franco A., Leekitcharoenphon P., Feltrin F., Alba P., Cordaro G., Iurescia M., Tolli R., D’Incau M., Staffolani M., Di Giannatale E. (2015). Emergency of a clonal lineage of multidrug-resistant ESBL-producing *Salmonella* Infantis transmitted from broilers and broiler meat to humans in Italy between 2011 and 2014. PLoS ONE.

[11] Hindermann D., Gopinath G., Chase H., Negrete F., Althaus D., Zurfluh K., Tall B.D., Stephan R., Nüesch-Inderbinen M. (2017). *Salmonella enterica* serovar Infantis from food and human infections, Switzerland, 2010–2015: Poultry-related multidrug resistant clones and an emerging ESBL producing clonal lineage. Front. Microbiol..

[12] Tate H., Folster J.P., Hsu C.H., Chen J., Hoffmann M., Li C., Morales C., Tyson G.H., Mukherjee S., Brown A.C. (2017). Comparative analysis of extended-spectrum-β-lactamase CTXM-65 producing *Salmonella enterica* serovar Infantis isolates from humans, food animals, and retail chickens in the United States. Antimicrob. Agents Chemother..

[13] Djeffal S., Bakour S., Mamache B., Elgroud R., Agabou A., Chabou S., Hireche S., Bouaziz O., Rahal K., Rolain J.M. (2017). Prevalence and clonal relationship of ESBL-producing *Salmonella* strains from humans and poultry in northeastern Algeria. BMC Vet. Res..

[14] Carfora V., Alba P., Leekitcharoenphon P., Ballaro D., Cordaro G., Di Matteo P., Donati V., Ianzano A., Iurescia M., Stravino F. (2018). Colistin resistance mediated by *mcr-1* in ESBL-producing, multidrug resistant *Salmonella* Infantis in broiler chickens industry, Italy (2016-207). Front. Microbiol..

[15] Velhner M., Kozoderović G., Grego E., Galić N., Stojanov I., Jelesić Z., Kehrenberg C. (2014). Clonal spread of *Salmonella enterica* serovar Infantis in Serbia: Acquisition of mutations in the topoisomerase genes *gyrA* and *parC* leads to increase resistance to fluoroquinolones. Zoonoses Public Health.

[16] Dionisi A.M., Lucarelli C., Benedetti I., Owczarek S., Luzzi I. (2011). Molecular characterization of multidrug-resistant *Salmonella enterica* serotype Infantis from humans, animals and the environment in Italy. Int. J. Antimicorb. Agents.

[17] Glynn M.K., Bopp C., Dewitt W., Dabney P., Mokhtar M., Angulo F.J. (1998). Emergency of multidrug-resistant *Salmonella* enterica serotype Typhimurium DT104 infections in the United States. N. Engl. J. Med..

[18] Le Hello S., Bekhit A., Granier S., Baura H., Beutlich J., Zaj M., Münch S., Sintchenko V., Bouchrif B., Fashae K. (2013). The global establishment of a highly fluoroquinolne resistant *Salmonella* enterica serotype Kentucky ST198. Front. Microbiol..

[19] Almeida F., Pitondo-Silva A., Oliveira M.A., Falcāo J.P. (2013). Molecular epidemiology and virulence markers of *Salmonella* Infantis isolated over 25 years in São Paulo State, Brazil. Infect. Genet. Evol..

[20] Chironna M., Tafuri S., Gallone M.S., Sallustio A., Martinelli D., Prato R., Germinario C. (2014). Outbreak of *Salmonella* Infantis gastroenteritis among people who had eaten at a hash house in southern Italy. Public Health.

[21] Ranjbar R., Rahmati H., Shokoohizadeh L. (2018). Detection of common clones of *Salmonella enterica* serotype Infantis from human sources in Tehran hospitals. Gastroenterol. Hepatol. Bed Bench.

[22] Sodagari H.R., Mohammed A.B., Wang P., O’Dea M., Abraham S., Robertson I., Habib I. (2019). Non-typhoidal *Salmonella* contamination in egg shells and content from retail in Western Australia: Serovar diversity, multilocus sequence types, and phenotypic and genomic characterizations of antimicrobial resistance. Int. J. Food Microbiol..

[23] Aghapour Z., Gholizadeh P., Ganbarov K., Bialvaei A.Z., Mahmood S.S., Tanomand A., Yousefi M., Asgharzadeh M., Yousefi B., Kafil H.S. (2019). Molecular mechanisms related to colistin resistance in Enterobacteriaceae. Infect. Drug Resist..

[24] Gunn J.S. (2008). The *Salmonella* PmrAB regulon: Lipopolysaccharide modifications, antimicrobial peptide resistance and more. Trends Microbiol..

[25] Sun S., Negrea A., Rhen M., Andersson D.I. (2009). Genetic analysis of colistin resistance in *Salmonella enterica* serovar Typhimurium. Antimicrob. Agents Chemother..

[26] Liu Y.Y., Wang Y., Walsh T.R., Yi L.X., Zhang R., Spencer J., Doi Y., Tian G., Dong B., Huang X. (2016). Emergence of plasmid-mediated colistin resistance mechanism MCR-1 in animals and human beings in China: A microbiological and molecular biological study. Lancet Infect. Dis..

[27] Cao Y.P., Lin Q.Q., He W.Y., Wang J., Yi M.Y., Lv L.C., Yang J., Liu J.H., Guo J.Y. (2020). Co-selection may explain the unexpectedly high prevalence of plasmid-mediated colistin resistance gene mcr-1 in a Chinese broiler farm. Zool. Res..

[28] Neuert S., Nair S., Day M.R., Doumith M., Ashton P.M., Mellor K.C., Jenkins C., Hopkins K.L., Woodford N., de Pinna E. (2018). Prediction of phenotypic antimicrobial resistance profiles from whole genome sequences of non-typhoidal *Salmonella enterica*. Front. Microbiol..

[29] Monte D.F., Lincopan N., Berman H., Cerdeira L., Keelara S., Thakur S., Fedorka-Cray P.J., Landgraf M. (2019). Genomic features of high-priority *Salmonella enterica* serovars circulating in the food production chain, Brazil, 2000–2016. Sci. Rep..

[30] Putman M., van Veen H.W., Konings W.N. (2000). Molecular properties of bacterial multidrug transporters. Microbiol. Mol. Biol. Rev..

[31] Kehrenberg C., Cloeckaert A., Klein G., Schwarz S. (2009). Decreased fluoroquinolone susceptibility in mutants of *Salmonella* serovars other than Typhimurium: Detection of novel mutations involved in modulated expression of *ramA* and *soxS*. J. Antimicrob. Chemoth..

[32] Salipante S.J., Hall B.G. (2003). Determining the limits of the evolutionary potential of an antibiotic resistance gene. Mol. Biol. Evol..

[33] Rehman M.A., Yin X., Persaud-Lachhman M.G., Diarra M.S. (2017). First detection of fosfomycin resistance gene, fosA7, in *Salmonella enterica* serovar Heidelberg isolated from broiler chickens. Antimicrob. Agents Chemother..

[34] Kadlec K., Pomba C.F., Couto N., Schwarz S. (2010). Small plasmids carrying *vga*(A) or *vga*(C) genes mediate resistance to lincosamides, pleuromutilins and streptogramin A antibiotics in methicillin-resistant *Staphylococcus aureus* ST398 from swine. J. Antimicrob. Chemoth..

[35] Schwarz S., Shen J., Kadlec K., Wang Y., Michael G.B., Feβler A.T., Vester B. (2016). Lincosamides, streptogramins, phenicols and pleuromutilins: Mode of action and mechanisms of resistance. Cold Spring Harb. Perspect. Med..

[36] Van Hoek A.H.A.M., Mevius D., Guerra B., Mullany P., Roberts A.P., Aarts H.J.M. (2011). Acquired antibiotic resistance genes: An overview. Front. Microbiol..

[37] Maguire F., Rehman M.A., Carrillo C., Diarra M.S., Beiko R.G. (2019). Identification of primary antimicrobial resistance drivers in agricultural nontyphoidal *Salmonella enterica* serovars by using machine learning. Clin. Sci. Epidemiol..

[38] Carattoli A., Bertini A., Villa L., Falbo V., Hopkins K.L., Threlfall E.J. (2005). Identification of plasmids by PCR-based replicon typing. J. Microbiol. Methods.

[39] Mc Millan E.A., Gupta S.K., Williams L.E., Jové T., Hiott L.M., Woodley T.A., Barrett J.B., Jackson C.R., Wasilenko J.L., Simmons M. (2019). Antimicrobial resistance genes, cassettes, and plasmids present in *Salmonella enterica* associated with United States food animals. Front. Microbiol..

[40] Szmolka A., Szabó M., Kiss J., Pászti J., Adrián E., Olasz F., Nagy B. (2018). Molecular epidemiology of the endemic multiresistance plasmid pSI54/04 of *Salmonella* Infantis in broiler and human population in Hungary. Food Microbiol..

[41] Azriel S., Goren A., Shomer I., Aviv G., Rahav G., Gal Mor O. (2017). The typhi colonization factor (Tcf) is encoded by multiple non-typhoidal *Salmonella* serovars but exhibits a varying expression profile and interchanging conribution to intestnal colonization. Virulence.

[42] Foley S.L., Johnson T.J., Ricke S.C., Nayak R., Danzeisen J. (2013). *Salmonella* pathogenicity and host adaptation in chicken associated serovars. Microbiol. Mol. Biol. Rev..

[43] Jiang L., Feng L., Yang B., Zhang W., Wang P., Jiang X., Wang L. (2017). Signal transduction pathway by the novel regulator LoiA low oxygen tension induced *Salmonella* Typhimurium invasion. PLoS Pathog..

[44] Elder J.R., Paul N.C., Burin R., Guard J., Shah D.H. (2018). Genomic organization of SPI1-3 in nutritional fitness of *Salmonella*. Int. J. Med. Microbiol..

[45] Muzzi A., Masignani V., Rappuoli R. (2007). The pan-genome: Towards a knowledge-based discovery of novel targets for vaccines and antibacterials. Drug Discov. Today.

[46] Goyal A. (2018). Metabolic adaptations underlying genome flexibility in prokaryotes. PLoS Genet..

[47] Clinical and Laboratory Standards Institute (2018). Methods for Dilution Antimicrobial Susceptibility Tests for Bacteria that Grow Aerobically.

[48] Clinical and Laboratory Standards Institute (2018). Performance Standards for Antimicrobial Susceptibility Testing.

[49] Schwarz S., Silley P., Simjee S., Woodford N., van Duijkeren E., Johnson A.P., Gaastra W. (2010). Editorial: Assessing the antimicrobial susceptibility of bacteria obtained from animals. Vet. Microbiol..

[50] Gwozdzinski K., Azarderakhsh S., Imirzalioglu C., Falgenhauer L., Chakraborty T. (2018). An improved medium for colistin susceptibility testing. J. Clin. Microbiol..

[51] (2013). EU Directive 2013/652/EU: Commission Implementing Decision on the Monitoring and Reporting of Antimicrobial Resistance in Zoonotic and Commensal Bacteria. https://eur-lex.europa.eu/legal-content/EN/TXT/?uri=CELEX%3A32013D0652.

[52] European Food Safety Authority, European Centre for Disease Prevention and Control (2019). The European Union summary report on antimicrobial resistance in zoonotic and indicator bacteria for humans, animals and food in 2017. EFSA J..

[53] Agersø Y., Torpdahl M., Zachariasen C., Seyfarth A., Hammerum A.M., Nielsen E.M. (2012). Tentative colistin cut-off value for *Salmonella* spp.. Foodborne Pathog. Dis..

[54] Andrews S. (2010). FastQC: A Quality Control Tool for High Throughput Sequence Data. http://www.bioinformatics.babraham.ac.uk/projects/fastqc.

[55] Liu B., Zheng D., Jin Q., Chen L., Yang J. (2019). VFDB 2019: A comparative pathogenomic platform with an interactive web interface. Nucleic Acids Res..

[56] Novović K., Mihajlović S., Dinić M., Malešević M., Miljković M., Kojić M., Jovčić B. (2018). *Acinetobacter* spp. porin Omp33-36: Classification and transcriptional response to carbapenems and host cells. PLoS ONE.

