# Supplementary material for: Genomic Characteristics of Colistin-Resistant Salmonella enterica subsp. enterica Serovar Infantis from Poultry Farms in the Republic of Serbia
Source: Antibiotics (Basel). 2020 Dec 10;9(12):886. doi: 10.3390/antibiotics9120886 (PMC7762970; doi:10.3390/antibiotics9120886)
Supplement: Supplementary file 1 [file antibiotics-09-00886-s001.zip › antibiotics-1006913-sm-SEND/Supplementary Tables and Figures/Supplementary Tables S3,4 and Figures S1,2,3.pdf]

Article

Genomic Characteristics of Colistin-Resistant *Salmonella enterica* subsp. *enterica* serovar Infantis from Poultry Farms in the Republic of Serbia

Branko Jovčić [1,2], Katarina Novović [1], Brankica Filipić [3], Maja Velhner [4,*], Dalibor Todorović [4], Kazimir Matović [5], Zoran Rašić [6], Sonja Nikolić [7], Ferenc Kiškarolj [8], and Milan Kojić [1]

1 Institute of Molecular Genetics and Genetic Engineering, University of Belgrade, Belgrade 11010, Serbia; bjovcic@bio.bg.ac.rs (B.J.); katarinanovovic@imgge.bg.ac.rs (K.N.), mkojic@imgge.bg.ac.rs (M.K.)
2 Faculty of Biology, University of Belgrade, Belgrade 11000, Serbia
3 Faculty of Pharmacy, University of Belgrade, Belgrade 11000, Serbia; brankica.filipic@pharmacy.bg.ac.rs
4 Scientific Veterinary Institute "Novi Sad", Novi Sad 21000, Serbia; dalibor@niv.ns.ac.rs
5 Veterinary Specialized Institute "Kraljevo", Kraljevo 36000, Serbia; matovic@vsikv.com
6 Veterinary Specialized Institute "Jagodina", Jagodina 35000, Serbia; vsij.rasic@gmail.com
7 Veterinary Specialized Institute "Niš", Niš 18000 Serbia; sonjavsinis@gmail.com
8 Veterinary Specialized Institute "Subotica", Subotica 24000, Serbia; kiskarolj.ferenc@vsisu.co.rs
* Correspondence: maja@niv.ns.ac.rs

Table S3. The CRISPR/Cas loci of seven colistin-resistant *S*. Infantis isolates detected using CRISPRCasFinder program.

Strain	Element	CRISPR Id/Cas type	Start	End	Spacer/Gene	Repeat consensus/*cas* genes
821	CRISPR	NODE_2_length_695018_cov_34_388291_1	207661	208177	8	CGGTTTATCCCCGCTGGCGCGGGGAACAC (-)
	Cas cluster	General-Class1	208274	576393	9	Cas1_0_IE, Cas2_0_IE, Cas3_0_IE, Cas3_0_IE, Cas5_0_IE, Cas6_0_IE, Cas7_0_IE, Cse1_0_IE, Cse2_0_IE
	CRISPR	NODE_2_length_695018_cov_34_388291_2	224329	224968	10	CGGTTTATCCCCGCTGGCGCGGGGAACAC (-)
92	CRISPR	NODE_3_length_204876_cov_26_23218	182238	182754	8	CGGTTTATCCCCGCTGGCGCGGGGAACAC (-)

		2_1				
	Cas cluster	General-Class1	182851	191304	8	Cas1_0_IE, Cas2_0_IE, Cas3_0_IE, Cas5_0_IE, Cas6_0_IE, Cas7_0_IE, Cse1_0_IE, Cse2_0_IE
	CRISPR	NODE_3_length_204876_cov_26_232182_1	198906	199545	10	CGGTTTATCCCCGCTGGCGCGGGGAACAC (-)
1371/1	CRISPR	NODE_3_length_526594_cov_23_616610_1	216751	218647	30	CGGTTTATCCCCGCTGGCGCGGGGAACAC (-)
	Cas cluster	General-Class1	218744	437702	9	Cas1_0_IE, Cas2_0_IE, Cas3_0_IE, Cas3_0_IE, Cas5_0_IE, Cas6_0_IE, Cas7_0_IE, Cse1_0_IE, Cse2_0_IE
	CRISPR	NODE_3_length_526594_cov_23_616610_2	234929	236545	26	CGGTTTATCCCCGCTGGCGCGGGGAACAC (-)
9181/1	Cas cluster	General-Class1	88893	307851	9	Cas1_0_IE, Cas2_0_IE, Cas3_0_IE, Cas3_0_IE, Cas5_0_IE, Cas6_0_IE, Cas7_0_IE, Cse1_0_IE, Cse2_0_IE
	CRISPR	NODE_3_length_416378_cov_28_024805_1	290050	291666	26	GTGTTCCCCGCGCCAGCGGGGATAAACCG (+)
	CRISPR	NODE_3_length_416378_cov_28_024805_2	307948	309844	30	GTGTTCCCCGCGCCAGCGGGGATAAACCG (+)
8418/2948	Cas cluster	General-Class1	88893	307851	9	Cas1_0_IE, Cas2_0_IE, Cas3_0_IE, Cas3_0_IE, Cas5_0_IE, Cas6_0_IE, Cas7_0_IE, Cse1_0_IE, Cse2_0_IE
	CRISPR	NODE_3_length_416378_cov_28_024805_1	290050	291666	26	GTGTTCCCCGCGCCAGCGGGGATAAACCG (+)
	CRISPR	NODE_3_length_416378_cov_28_024805_2	307948	309783	29	GTGTTCCCCGCGCCAGCGGGGATAAACCG (+)
9520/2	CRISPR	NODE_4_length_416384_cov_17_646440_1	106535	108431	30	CGGTTTATCCCCGCTGGCGCGGGGAACAC (-)
	Cas cluster	General-Class1	108528	327492	9	Cas1_0_IE, Cas2_0_IE, Cas3_0_IE, Cas3_0_IE, Cas5_0_IE, Cas6_0_IE, Cas7_0_IE, Cse1_0_IE, Cse2_0_IE
	CRISPR	NODE_4_length_416384_cov_17_646440_2	124713	126329	26	CGGTTTATCCCCGCTGGCGCGGGGAACAC (-)
3842	CRISPR	NODE_3_length_332755_cov_41_01606	221693	222392	11	CGGTTTATCCCCGCTGGCGCGGGGAACAC (-)

		0_1				
	Cas cluster	General-Class1	222368	224440	1	Cas3_0_IE
	CRISPR	NODE_3_length_332755_cov_41_016060_2	232155	233465	21	CGGTTTATCCCCGCTGGCGCGGGGAACAC (-)

Table S4. Primers used in this study.

Primer	Sequences	Product size (bp)	Reference
***phoP* Fw**	5′ GATACGCCGCTGGCGCAGCAG 3′	147	This study
***phoP* Rev**	5′ CGCGCCATTACCTCTTCGATG 3′		
***phoQ* Fw**	5′ CCGGAGACGACGCGTGAGCTG 3′	147	
***phoQ* Rev**	5′ CGTACTCTGCAAAACCGCGAG 3′		
***mgrB* Fw**	5′ CGATGGGTCGTTCTCGGCATC 3′	111	
***mgrB* Rev**	5′ GATGGCGCAAATACCGCTG 3′		
***pmrA* Fw**	5′ CCCTGCCGGTACTCATTCTG 3′	154	
***pmrA* Rev**	5′ CCCTGGTTATTATGGCGGC 3′		
***pmrB* Fw**	5′ CCGTCGTCTCGGCGATCAATC 3′	164	
***pmrB* Rev**	5′ CGGCGCGACATCAACATTGTG 3′		
***gyrB* Fw**	5′ GGTCTGCCGGGCAAACTGG 3′	149	
***gyrB* Rev**	5′ GAGGATTTTACCTTTCAGCGGCAG 3′		

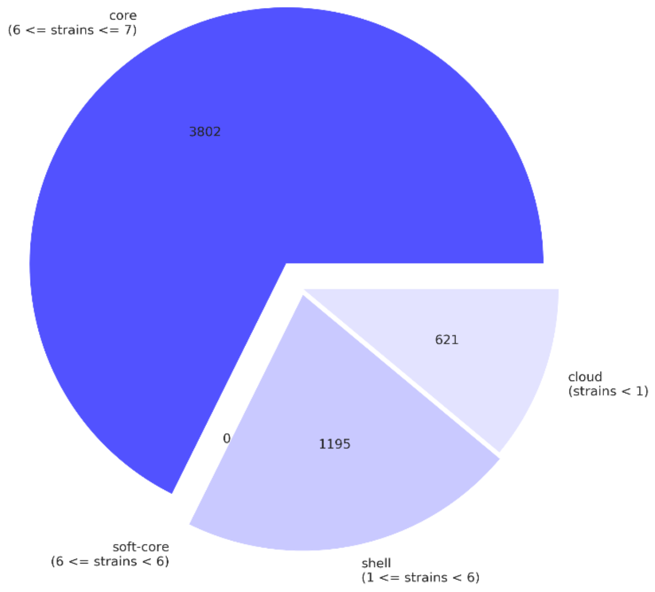

Figure S1. Distribution of the core and accessory genes within pan-genome.

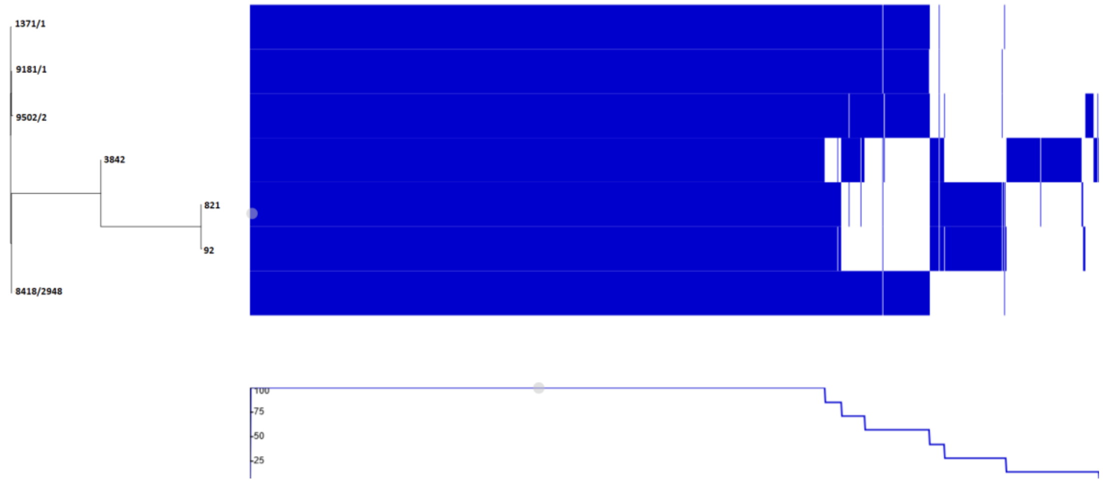

Figure S2. Visualization of pan-genome constructed using Roary based on the core and accessory genes showing phylogenetic relatedness of the isolates by blue (present) and white (absent) fragments. At the bottom of the figure the percentage of strains that have each gene is shown.

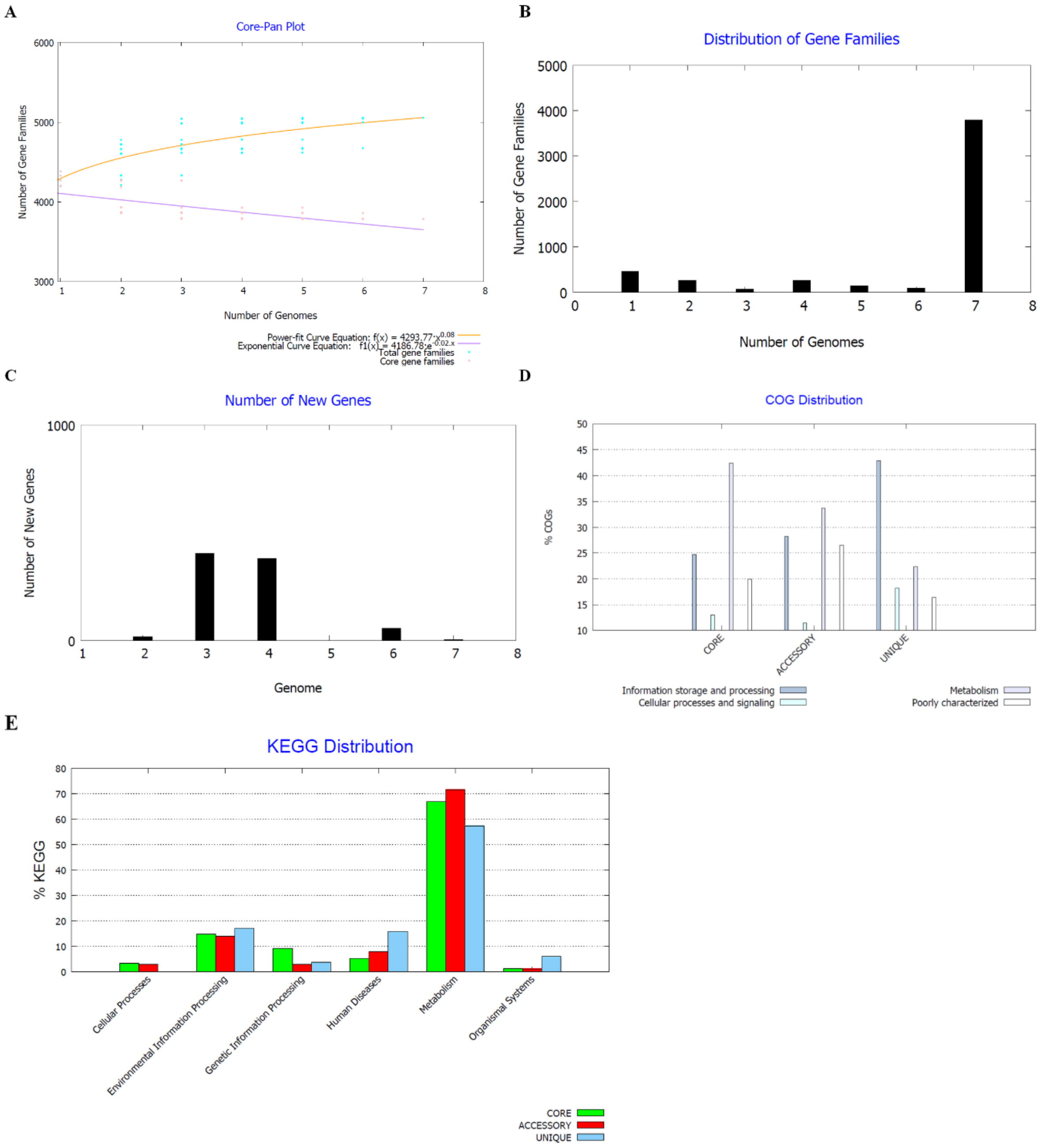

Figure S3. Pan-genome analysis: pan and core genome curves (A); frequency distribution of gene families within genomes (B); the number of new genes added to each genome (C); the COG and KEGG distribution of the representative proteins in the core, accessory, and unique genome (D and E).
